# Assessing pattern of the Pediatric Multisystem Inflammatory Syndrome (PMIS) in children during the COVID-19 pandemic: experience from the emergency department of tertiary care center of a low-middle-income country

**DOI:** 10.1186/s12887-024-04572-x

**Published:** 2024-02-03

**Authors:** Saleem Akhtar, Iqra Anis, Nirdosh Ashok Kumar, Muhammad Tayyab Ihsan, Ahmed Raheem, Surraiya Bano

**Affiliations:** 1 Department of Pediatrics and Child Health, Aga Khan University Hospital, Karachi, Pakistan; 2 Department of Emergency Medicine, Aga Khan University Hospital, Karachi, Pakistan; 3 Aga Khan Medical College, Karachi, Pakistan

**Keywords:** Pediatric Multisystem Inflammatory Syndrome, COVID-19, Emergency Department

## Abstract

**Background:**

Pediatric Multisystem Inflammatory Syndrome (PMIS) is a hyperinflammatory condition affecting multiple organs in children, often resembling incomplete Kawasaki Disease during later phases of COVID-19 infection. Data on PMIS in low-middle-income countries, particularly in emergency department settings, is limited.

**Objectives:**

This prospective observational study at Aga Khan University Hospital, Karachi, aimed to determine the frequency, clinical presentation patterns, and laboratory parameters of children with PMIS visiting the emergency department during the COVID-19 pandemic. Secondary objectives included assessing factors associated with in-hospital mortality.

**Methods:**

From March 2020 to September 2021, patients meeting World Health Organization PMIS criteria were enrolled. COVID-19 testing included PCR and antibody testing. Data was collected through a questionnaire and analyzed statistically.

**Results:**

Among 56 PMIS patients (85.7% male, mean age 7.67 ± 4.8 years), respiratory symptoms (70%), neurological symptoms (57%), and gastrointestinal symptoms (54%) were common presentations. Signs included delayed capillary refill time (93%), low-volume pulses (89%), and hypotension (68%). COVID-19 antibodies were positive in the majority (78.6%) while PCR was positive in 18%. Risk factors for mortality included prolonged emergency department stay, and high Ferritin and Lactate Dehydrogenase levels.

**Conclusion:**

PMIS affects children of all ages. Respiratory and gastrointestinal symptoms are the most frequent presentations. Elevated inflammatory markers, including LDH, Ferritin, D-dimer, and Pro-BNP, correlate with higher mortality risk.

## Introduction

The 2019 novel coronavirus disease (COVID-19) caused by severe acute respiratory syndrome coronavirus 2 (SARSCoV2) is a viral pandemic that spread worldwide. During the early phase of the COVID-19 pandemic, children were thought to be less affected, with mild respiratory symptoms and low morbidity and mortality [1, 2]. As the pandemic progressed, several studies reported severe complications in children with features of significant inflammation, toxic shock syndrome, and clinical features similar to incomplete Kawasaki Disease (KD) [3]. In particular, a syndrome of hyperinflammatory process associated with fever emerged in the pediatric population with positive COVID-19 test results [4]. The syndrome was later described as Pediatric Inflammatory Multisystem Syndrome (PMIS) by the Royal College of Pediatrics and Child Health (RCPCH) and as Multisystem Inflammatory Syndrome in Children (MIS-C) by the World Health Organization (WHO) and Centers for Disease Control and Prevention (CDC) [5–7]. PMIS or MISC was preliminarily defined as a hyperinflammatory syndrome with multiorgan involvement and clinical features that overlap with KD [8].

The cases of this hyperinflammatory syndrome have similarities to KD and Toxic Shock Syndrome (TSS) [9]. Several cases have been reported from the emergency department (ED) with shock and multiorgan failure. However significant data is lacking, especially from the emergency departments (EDs) of low-middle-income countries (LMIC) [10]. This study was conducted in an ED of a tertiary care hospital in a low-middle-income country to fulfill this research gap. This study aims to determine the frequency, pattern of presentations, and laboratory parameters in children presenting with Pediatric Multisystem Inflammatory Syndrome (PMIS) to ED during the COVID-19 pandemic.

## Methods

### Study setting and duration

This prospective observational study took place in the Emergency Department (ED) of Aga Khan University Hospital (AKUH) located in Karachi, Pakistan. The study spanned from March 2020 to September 2021. AKUH’s ED is equipped with 60 beds, including 23 dedicated to pediatric patients. On an annual basis, the ED typically sees around 15,000 pediatric patients, and it maintains continuous faculty coverage, ensuring 24/7 medical care availability.

### COVID-19 screening

Given the study’s timeframe during the COVID-19 pandemic, all patients presenting with fever and respiratory symptoms underwent thorough COVID-19 screening. Multiple case definitions and guidelines, as recommended by reputable entities like the World Health Organization (WHO), Centers for Disease Control and Prevention (CDC), and the Royal College of Paediatrics and Child Health (RCPCH), were considered. In particular, we strictly adhered to the WHO’s criteria for case definition to maintain methodological consistency throughout the study.

### Pediatric Multisystem Inflammatory Syndrome (PMIS)

Children exhibiting clinical indications of Pediatric Multisystem Inflammatory Syndrome (PMIS), according to the preliminary definition criteria established by the WHO, were included in the screening process. In cases where the initial COVID-19 Polymerase Chain Reaction (PCR) test yielded negative results but symptoms strongly suggested PMIS, subsequent COVID-19 antibody testing was conducted to further assess their COVID-19 status.

### Data collection

For data collection, a meticulously prepared questionnaire was employed. This questionnaire was thoughtfully designed to systematically capture pertinent information, including patient demographics (age, gender), chief presenting complaints (e.g., fever, respiratory distress), investigations performed (e.g., laboratory tests, imaging), treatments administered (e.g., medications, interventions), duration of the patient’s stay in the ED, duration of their hospital stay, and any recorded mortalities. This method of data acquisition was implemented to ensure a comprehensive and standardized approach, ultimately enhancing the reliability and robustness of the findings derived from this study.

### Statistical analysis

The process of data management was conducted utilizing Microsoft Excel Spreadsheet (2010), followed by the seamless transition of the refined dataset into SPSS-22 (IBM, IL, USA) for comprehensive statistical scrutiny. To provide a coherent overview of the data, we employed descriptive statistics. This involved presenting numerical counts with accompanying percentages for qualitative variables, while continuous variables were expressed as either mean ± standard deviation or medians accompanied by their respective ranges. The comparative assessment of in-hospital mortality against triage vitals, encompassing parameters such as Systolic Blood Pressure (SBP), Diastolic Blood Pressure (DBP), Heart Rate (HR), and Respiratory Rate (RR), was executed through the application of the Mann-Whitney U test.

For the investigation of associations between distinct groups (survivors vs. deceased), categorical data underwent scrutiny via either the Chi-square test or Fisher’s exact test. In instances of normally distributed continuous outcomes, the t-test was employed for comparison purposes. Employing a multifaceted approach, multivariate analysis was engaged to unveil potential risk factors influencing mortality.

Significance within our analyses was denoted by a *p*-value equal to or less than 0.05. The focal point of our study, the in-hospital mortality rate, was addressed as the primary endpoint. The presentation of results involved rendering Odds Ratios (OR) accompanied by their corresponding 95% confidence intervals, ensuring a comprehensive portrayal of the statistical findings with a level of precision that aids in informed interpretation.

## Results

A total of 56 patients were diagnosed with PMIS. The mean age of the patients was 7.67 ± 4.8 years, ranging from 1 month to 16 years. Notably, all age groups, encompassing infants, children, and adolescents, were affected with equal frequency. Our study exhibited a distinct male predominance, accounting for 85.7% of the cases. A comprehensive depiction of patients’ demographics and baseline characteristics is available in Table 1.

**Table 1:** Demographic & Baseline Characteristics of Pediatric Multisystem Inflammatory Syndrome

	Mean ± .D.S.D. (Range)	
Age in years	7.67 (± 4.8) (1–16)	
Weight in Kg	22.46 (± 17.1) (3.5–82)	
**Age Groups**	**Percentage (%)**	**Frequency (f)**
1 month to 1 Years	30.40%	(17)
2–5 Years	19.60%	(11)
6–10 Years	23.20%	(13)
11–16 Years	26.80%	(15)
**Gender**		
Female	14.30%	(8)
Male	85.70%	(48)
**E.D length of stay**		
Mean ± .D.S.D. (Range: Min-Max)	5.41 ± 3.10 (1.5 to 16.5 h)	
< 4 hrs	25.00%	(14)
>4 hrs	75.00%	(42)
**Hospital Length of Stay**		
Mean ± .S.D. (Range: Min-Max)	7.14 ± 5.97 (1 to 30 Days)	
< 5 Days	37.50%	(21)
>5 Days	62.50%	(35)
**Status**		
Alive	82%	(46)
Expired	18%	(10)

The chief presenting complaints encompassed respiratory, neurological, gastrointestinal, and dermatological manifestations. Clinical assessments revealed prevalent signs including delayed capillary refill time (93%), low volume pulses (89%), hypotension (68%), and tachycardia (60%). Further details regarding presenting complaints and examination findings are outlined in Table 2.

**Table 2:** Presenting complaints and examination findings

General and Physical examination and Cardiac parameters		
**Presenting Complaints**	Percentage(%)	Frequency
Respiratory symptoms	69.60%	(39)
GI Symptoms	53.60%	(30)
Skin Manifestations	33.90%	(19)
Neurological manifestations	57.10%	(32)
**General Physical Examination**		
Cervical lymphadenopathy	10.70%	(6)
Non Purulent Conjunctivitis	16.10%	(9)
Strawberry tongue	28.60%	(16)
Skin Manifistations	33.90%	(19)
Pedal edema	3.60%	(2)
**Cardiac Examination**		
Tachycardia	60.70%	(34)
Hypotension	67.90%	(38)
** Vitals**	**Mean ± SD (Range: Min-Max)**	
Systolic Blood Pressure (SBP)	86.96 (± 15.4) (50–110)	
Diastolic Blood Pressure (DBP)	49.11 (± 12.5) (30–76)	
Heart Rate (HR)	137.29 (± 28.3) (84–221)	
Respiratory Rate (RR)	33.16 (± 9.8) (20–60)	
Temperature	37.3 (± 2.2) (27–40)	
Glasgow Coma Scale (GCS)	13.05 (± 3.2) (3–15)	
**Capillary Refill time (CRT)**	Percentages (%)	Frequency
< 2 s	7%	(4)
>2 s	93%	(52)
**Pulses**		
Low volume	89%	(50)
Normal volume	11%	(6)
**Systolic Function**		
Normal	55%	(31)
Mild dysfunction	14%	(8)
Moderate dysfunction	16%	(9)
Severe dysfunction	14%	(8)
**Electrocardiogram (ECG)**		
Sinus Tachycardia	91%	(51)
Ventricular Tachycardia	2%	(1)
Supraventricular Tachycardia	4%	(2)
Normal Sinus Rythm	4%	(2)

Our screening protocol involved COVID-19 evaluation for all 56 patients. Initially, a COVID-19 PCR test was conducted, followed by COVID-19 antibodies if PCR yielded negative results. Interestingly, COVID-19 PCR testing yielded positive results in only 18% (10) of the patients, whereas COVID-19 antibodies were detected in 78.6% (44). Among the 12 patients with negative COVID-19 antibodies, 83.3% (10) tested PCR positive, with a mere 16.7% (2) displaying both negative antibodies and PCR results.

Hematological, inflammatory biomarkers and biochemical parameters were assessed, and their descriptive characteristics are presented in Table 3. Comprehensive cardiac evaluations, inclusive of Electrocardiography (ECG), Troponin, N-type Pro BNP, and Transthoracic Echocardiography, were performed for all patients. Sinus tachycardia with non-specific ST-T changes dominated ECG findings (91%), with only a minimal percentage exhibiting ventricular tachycardia (2%), supraventricular tachycardia (4%), or a normal sinus rhythm (4%).

**Table 3:** Hematological, inflammatory, and biochemical markers in patients with PMIS

Parameters	Mean ± .D.S.D. (Range)
**Inflammatory Markers**	
C reactive protein	135.37 (± 99.6) (0.37-383.59)
ESR	46.09 (± 23.2) (2–74)
Ferritin	2497.6 (± 3881.1) (76.4-17729)
LDH	945.22 (± 1652.4) (219-10176)
**Cardiac Marker**	
Troponin I	25.06 (± 115.5) (0.01–739)
Pro_BNP	38,194 (± 79957.9) (75-376052)
**Hematological Parameters**	
Hemoglobin	10.2 (± 1.7) (6.9–13.5)
Total Leukocyte count	13.12 (± 6.7) (1.6–26.3)
Neutrophils	69.11 (± 20.1) (10.7–94.4)
Lymphocytes	23.73 (± 17.7) (3.3–83.7)
Platelet counts	217.02 (± 160.6) (15–709)
**Biochemistry Parameters**	
Serum Procalcitonin	34.01 (± 41.6) (0.12–100)
Serum Sodium	137.11 (± 9.1) (125–172)
Serum Potassium	4.08 (± 1.4) (1.8–9.8)
Serum Chloride	102.75 (± 8.6) (92–139)
Serum Bicarbonate	18.94 (± 5.9) (5.1–30.7)
Serum Lactate	4.65 (± 3.6) (0.7–14)
Blood Urea Nitrogen	25.56 (± 16.4) (5–94)
Serum Creatinine	0.81 (± 0.5) (0.3–2.4)
**Coagulation profile**	
PT	18.77 (± 22.9) (10.1–170)
APTT	41.49 (± 30.3) (24.7–170)
INR	1.83 (± 2.3) (0.9–17)
D DIMER	13.32 (± 11.8) (0.3–30)
**COVID-19 Antibodies**	
Non Reactive	(12) 21.4%
Reactive	(44) 78.6%
**COVID − 19 PCR**	
Negative	(46) 82.1%
Positive	(10) 17.9%

Echocardiography results showcased a spectrum of left ventricular systolic dysfunction: 55% exhibited normal function, while 45% displayed variable degrees of dysfunction. Troponin elevation was noted in 70% of patients, with a raised N-type Pro BNP level observed in 78% of cases.

Therapeutically, 78–85% of patients received Intravenous Immunoglobulin (IVIG) and methylprednisolone as anti-inflammatory agents. Epinephrine was administered solely to 68% of patients for inotropic support, while the remaining individuals received a combination of Epinephrine with either Milrinone or Norepinephrine infusion. Respiratory assistance was requisite for 75% of patients, with 41% employing non-invasive methods like High Flow Nasal Cannula (HFNC), and 35% necessitating invasive interventions such as endotracheal intubation and mechanical ventilation.

The multivariate binary regression model unveiled several notable risk factors linked to mortality. These findings are summarized in Table 4.

**Table 4:** Univariate and multivariate binary regression analysis for the prediction of mortality

Factors	Univariate		Multivariate	
	OR [95% CI]	*P*-value	OR [95% CI]	*P*-value
Male gender	0.6 [0.1–3.53]	0.572		
Dialysis (Yes)	7 [1.37–35.68]	0.019*		
Hypotension (Yes)	4.25 [1.02–17.69]	0.047*		
Tachycardia (Yes)	6.22 [1.19–32.68]	0.031*		
ED Stay > 4 h	5.4 [1.27–22.93]	0.022*	15.75 [1.25–198.5]	0.033*
Platelet level > = 150	3.17 [0.72–13.87]	0.126		
Ferritin level ( > 322 ng/mL)	14 [10.6 -56.33]	0.017*	23.5 [2.5–61.3]	0.011*
Pro BNP (> 1000 pg/ml)	9.82 [1.15–83.91]	0.037*		
Troponin level (> 0.006 ng/ml	3.39 [0.37–31.33]	0.282		
Epinephrine (Yes)	1.13 [0.26–4.99]	0.873		
Epinephrine & Milrinone (Yes)	1.88 [0.47–7.45]	0.372		
Epinephrine & Norepinephrine (Yes)	4.25 [1.02–17.69]	0.047*		
D-Dimer level (> 0.5 µ/mL)	3.49 [1.54–18.66]	0.032*	2.75 [1.86–40.53]	0.046*
LDH level (> 246 U/L)	9 [2.85–56.7]	0.045*	20.5 [3.85–53.6]	0.024*
PT level (> 12)	8.67 [1.59–47.15]	0.012*		
INR level	11.2 [2.03–61.89]	0.006*		
Lactate level ( > 4 mmol/L)	1.29 [0.29–5.68]	0.739		

*Abbreviations: C.I, Confidence interval, OR, Odd ratios*

*Univariate and multivariate logistic regression for the prediction of mortality*

*(International Normalized Ratio = INR, PT = prothrombin Time, ED = Emergency, LDH = Lactate dehydrogenase, BNP = B-type natriuretic peptide*

## Discussion

Initially, COVID-19 in children appeared mild. However, a subset of cases exhibited severe inflammation and multi-organ problems, leading to Pediatric Inflammatory Multisystem Syndrome (PMIS), or Multisystem Inflammatory Syndrome in Children (MIS-C). This study investigates the frequency, presentation, and lab findings in PMIS cases among children, offering scientific insights into this condition.

In this study, we examined 56 pediatric patients diagnosed with Pediatric Inflammatory Multisystem Syndrome (PMIS) in the emergency department (ED) at Aga Khan University Hospital (AKUH), Pakistan. Notably, our study highlights several key findings. We observed a gender and age distribution in PMIS cases that differs from previous reports. Our cohort had a higher mean age (7.67 years) compared to studies from Latin America, France, and Switzerland. Males represented most cases (85%), aligning with existing literature suggesting gender-associated risk factors [13–16].

Our study revealed differences in clinical presentation compared to studies in other regions. Most of our patients presented with respiratory complaints (70%), followed by neurological symptoms (57%). In contrast, studies from the U.S. reported gastrointestinal symptoms (80%) and cardiovascular issues, including echocardiographic anomalies and hypotension (63%). Regardless of the region, a significant proportion of PMIS cases required intensive care [14].

Interestingly, gastrointestinal complaints were notably prevalent in the group of patients who did not survive in our sample. In our cohort, respiratory complaints dominated as the most frequent patient grievance, followed by neurological issues. This diverges from a U.S. study encompassing 26 states, wherein gastrointestinal involvement was reported in 92% of cases, whereas respiratory symptoms accounted for 70% of the sample. Another systematic review of MIS-C outlined that 4 out of 5 cases exhibited diarrhea and abdominal pain, at times severe enough to mimic appendicitis. This might be attributed to SARS-CoV-2’s mode of transmission, impacting angiotensin-converting enzyme receptors (ACE receptors) prevalent in the gastrointestinal tract [23]. Given children’s underdeveloped hand hygiene habits and relatively immature immune systems, it’s plausible that gastrointestinal symptoms are more pronounced, particularly as the virus establishes itself in the body after clearing from the upper respiratory tract [21].

All patients in our study underwent COVID screening, with most displaying positive serology results, alongside some showing positive PCR outcomes. This aligns with the fact that PMIS typically manifests weeks after the active viral infection, rendering PCR positivity obsolete and antibodies crucial. This trend supports the hypothesis that PMIS could stem from the immune response against the virus, potentially triggering a cytokine storm and culminating in hyperinflammation. Severe cases might involve antibody-dependent enhancement, where certain antibodies fail to neutralize the virus, inadvertently facilitating its propagation [25, 13].

The majority of our study cohort presented with abnormal ECG readings, a hallmark of myocarditis due to its frequent association with sinus tachycardia, a common finding in myocarditis. Multiple studies have highlighted the connection between COVID-19 and myocarditis-like symptoms, often characterized by arrhythmias [26, 24]. Similarly, our findings emphasized sinus tachycardia, accompanied by ventricular tachycardia, implicating cardiac involvement. Consequently, most patients demonstrated delayed capillary refill time and required cardiac resynchronization therapy in under 2 s.

Notably, inflammatory markers indicative of cardiac injury, such as ESR, ferritin, LDH, fibrinogen, and CRP levels, were elevated in patients fulfilling PMIS criteria [26]. This corresponds to the underlying pathogenesis, wherein our study observed elevated LDH, Ferriten, D-dimer, and Pro-BNP levels associated with a higher risk of mortality. Particularly, patients with LDH levels exceeding a certain threshold displayed a pronounced association with the group of patients who did not survive [25]. Although the number of fatalities post-admission to the ED remained low in our sample [18, 19]. All patients were admitted to the Pediatric intensive care unit, required vasopressors, non-invasive and some required invasive mechanical ventilation [17]. As far as immunosuppressive therapy is concerned, the majority were treated with steroids and intravenous immunoglobulin. This is consistent with the studies by Belhadjer Z et al. who have reported the use of immunoglobulin in all their patients while adjunctive steroids were used in 3rd of their patient population [11].

It’s important to acknowledge the limitations of our study. The data exclusively originated from within AKUH, thereby constraining the sample size. Our data collection and analysis methods omitted geographical and sociocultural diversity comparisons. Moreover, the diagnosis of COVID-19 relied solely on throat PCR, as alternative methods like stool PCR were unavailable within our facilities. This approach, while valuable for detecting SARS-CoV-2 after clearance from the upper respiratory tract, has its limitations.

In summary, our study adds to the growing body of knowledge concerning PMIS in pediatric populations, offering insights into its prevalence, presentation, and laboratory profiles. By examining cases in a developing country like Pakistan, we contribute to a more comprehensive understanding of this complex condition and underscore the global significance of its clinical manifestations.

## Conclusion

PMIS affects children of all ages, with a male predominance. Respiratory and gastrointestinal symptoms are the most frequent presentations.Myocarditis is the most common cardiac manifestation. Elevated inflammatory markers, including LDH, Ferritin, D-dimer, and Pro-BNP, correlate with higher mortality risk.

Our collective insights will guide clinical management and public health responses to PMIS in children during the ongoing pandemic. Further research across diverse settings will deepen our understanding and address the challenges posed by PMIS.

## Data Availability

The datasets used and/or analyzed during the current study are available from the corresponding author upon reasonable request.
